# A Comparative Review of Water Flossers in Periodontal Therapy

**DOI:** 10.7759/cureus.50162

**Published:** 2023-12-08

**Authors:** Abdulaziz M Altalhi, Nadeen S Alqahtani, Jehan A Alareefi, Shahad S Alamri, Khalid S Almutairi, Razan A Bin Dous, Ibrahim A Albaqami

**Affiliations:** 1 Dentistry, Ministry of Health, Riyadh, SAU; 2 Dentistry, Ministry of Health, Makkah, SAU; 3 Dentistry, Ajmal Ebtesama, Buraydah, SAU; 4 General Dentistry, Vision Colleges, Jeddah, SAU; 5 General Dentistry, Qassim University, Buraydah, SAU; 6 General Dentistry, Kadoon Dental Clinic, Riyadh, SAU

**Keywords:** plaque control, oral irrigation, water floss, oral hygiene instruction (ohi), periodontal disease

## Abstract

This study investigated the efficacy of water flossers (WFs), devices used to irrigate the interdental and subgingival areas, compared to other interdental care methods, in the management of periodontal disease. A computerized PubMed search was conducted by the author, encompassing the years 1962 to 2023. The year 1962 was selected due to it being the introduction of the first WF. Keywords included "oral irrigator", "efficacy," and "water flossers." The review provided a broad comparative assessment of WFs, rather than an exhaustive detailed article review. We discussed the history and evolution of commercially available WFs and introduced a novel classification system. The research also evaluated the performance of WFs in comparison to traditional and novel interdental care methods, focusing on crucial clinical parameters such as plaque removal efficiency and reduction in gingival inflammation. The results of the study reveal that WFs appear to be superior in the management of periodontal disease and have demonstrated effectiveness in a variety of indices. However, it's worth noting that the author did not statistically analyze any of the data. We identified gaps in the literature and found opportunities for further clinical studies. These findings hold implications for optimal periodontal disease prevention and management, addressing the evolving landscape of oral care practices.

## Introduction and background

Periodontal diseases are pathological processes that involve the periodontium, the supportive apparatus surrounding the tooth. This apparatus includes the gingival tissue, alveolar bone, cementum, and periodontal ligament [1]. The inflammatory response to infection by periodontal pathogens is the primary cause of periodontal destruction. The effectiveness of disease management requires understanding associated risk factors such as diabetes, smoking, age, socioeconomic status, and chronic diseases like osteoporosis, cardiovascular disease, and, importantly, dental and interdental hygiene [2]. To reduce the occurrence of periodontal disease, mechanical interventions such as scaling and root planing, combined with proper oral hygiene measures that include the use of interdental aids, can effectively control periodontal diseases. This combined approach may subsequently reduce the risk of related systemic conditions and enhance periodontal health outcomes [3].

Interdental aids commonly aim to clean between teeth and remove plaque and debris. These aids encompass practices such as interdental brushing and flossing and are a crucial component in the prevention of periodontitis and caries. Common traditional interdental care methods include flossing with dental floss, interdental brushes, interdental tape, electric flossers, and wooden toothpicks. Over the past decade, water flossers (WFs), also known as oral irrigators or dental water jets, are devices designed to improve periodontal health by directing a pressurized stream of water at the teeth and interdental area, removing supragingival and subgingival food particles, pathogenic bacteria, and plaque [4]. Research has demonstrated that incorporating medicaments into the water tank of WFs during their use can significantly enhance treatment efficacy for patients with periodontitis, leading to improved overall periodontal health outcomes [5,6].

We summarized the existing literature and provided a comprehensive review of studies investigating the impact of WFs on periodontal disease management. We provided historical context into WFs, created a novel classification system of WFs, and evaluated and compared its efficacy as opposed to traditional interdental aids. We highlighted gaps and limitations in the current understanding of the efficacy of WFs in managing periodontal disease, to identify areas for future research. This review also looks ahead to the future applications of WFs in periodontal disease management and discusses its potential in individualized patient care.

## Review

Research methodology

An extensive search was conducted across various electronic databases to gather scholarly articles pertinent to the efficacy of WFs in periodontal disease management. The databases accessed for this comprehensive literature review included PubMed, Embase, Medline, and Google Scholar. The search strategy was meticulously designed, utilizing specific keywords related to WFs and their role in periodontal health. These keywords were carefully chosen to capture the broad spectrum of existing research on this topic.

The inclusion criteria for selecting articles were precise. The literature had to be published in English and needed to be available in full-text format. This review encompassed a diverse range of study types, including prospective or retrospective studies, randomized controlled trials, case series, case reports, and review papers. The goal was to provide a holistic understanding of the current state of knowledge regarding WFs in the context of periodontal disease management.

**Figure 1:**
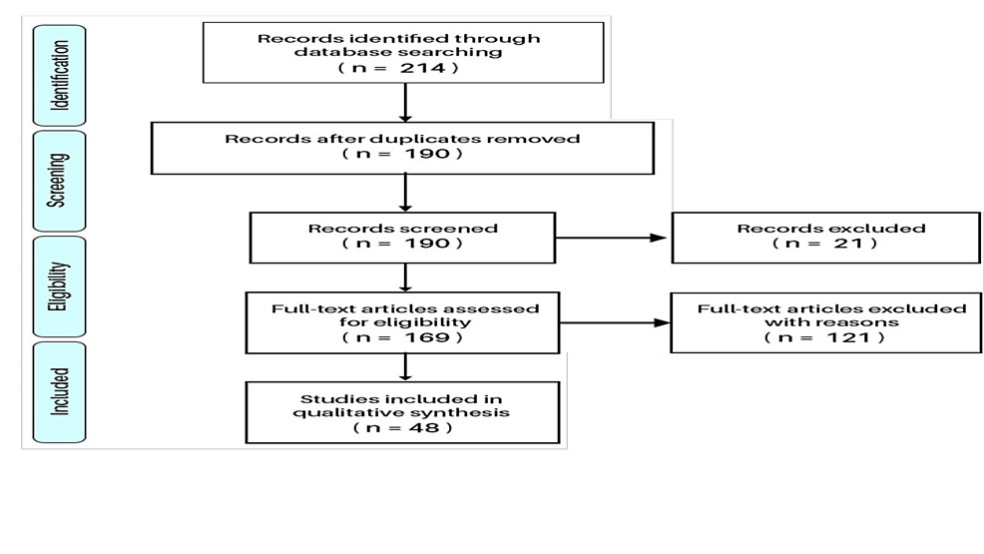
Selection process for studies on the efficacy of water flossers in periodontal disease management. Image credit: Abdulaziz M. Altalhi.

The exclusion criteria included non-English language studies, research not specifically addressing WFs' efficacy in managing periodontal disease, and studies beyond the scope of human clinical trials. Titles and abstracts not pertinent to WF use in periodontal disease were also omitted to ensure focused and relevant results. Figure 1 presents the article selection process for our comprehensive review.

History of WFs

WFs have gained popularity in oral hygiene routines due to their effectiveness in removing plaque and promoting gingival health. These devices utilize a pulsating stream of water to clean between teeth and along the gingiva, offering an alternative to traditional flossing methods. The concept of oral irrigation dates back to the early 1960s when Dr. Gerald Moyer and John Mattingly developed early versions of WFs. In 1962, the first commercially available WF, known as Water Pik, was introduced. It marked a significant step in making oral irrigation accessible to the public [7].

In 2001, the American Academy of Periodontology recognized the clinical benefits of water flossing, stating that “supragingival irrigation with or without medicaments reduces gingival inflammation beyond what is normally achieved by toothbrushing alone” [8].

Mechanism of action of WFs

WFs operate based on a combination of pressure and pulsation mechanisms. The device typically consists of a reservoir for water and a hand-held nozzle. As the user directs the nozzle between their teeth and along the gumline, a pressurized stream of water is expelled. WFs may be traditional, power-driven, clinical grade, portable, or adaptive in nature. We introduce a novel classification for WFs, based on their application (Table 1).

**Table 1:** Novel classification system for WFs based on their application.

Classification	Type of water flosser (WFs)	Description
Class 1	Traditional	These are standard WFs with a water reservoir and a nozzle for directing water between teeth and along the gumline.
Class 2	Power-driven	This category includes electric or power-driven WFs that provide additional features such as adjustable pressure settings and pulsating water streams for enhanced cleaning.
Class 3	Clinical grade	Specifically designed for professional dental use, clinical-grade WFs may have advanced features for precise control and comprehensive cleaning.
Class 4	Portable	Compact and travel-friendly, portable WFs are designed for on-the-go use, often powered by batteries or USB charging.
Class 5	Adaptive	Advanced models may incorporate smart technology, adapting to individual oral care needs, providing real-time feedback, and optimizing cleaning routines.

The effectiveness of WFs in dental care, particularly in managing periodontal disease, is underscored by several key mechanisms and benefits. Initially, WFs are characterized by two zones of hydrokinetic activity: an impact zone at the gingival margin and a flushing zone where the irrigant reaches beneath the gum margin. This specific action enables the elimination of supragingival plaque and the clearing of subgingival bacteria and other debris [7,9].

Furthermore, WFs have demonstrated a capability to reduce inflammation by lowering pro-inflammatory cytokines (IL-1β and PGE2) in the gingival crevicular fluid, particularly benefiting individuals with mild-to-moderate periodontitis and diabetes [10,11]. In terms of penetration and effectiveness, studies have shown that WFs can effectively penetrate 90% of pockets with probing depths of 6 mm or less [12], significantly reducing periodontal pathogens up to these depths without harming soft tissues. Similar observations were made by Cobb et al. [13], who found that WFs reduced periodontal pathogens up to pocket depths of 6 mm. Even in deeper pockets (≥7 mm), WFs have been found to reach up to 68% of the periodontal pockets [14].

The pulsating action of WFs induces a qualitative change in subgingival plaque and is considered safe for the soft tissues in the mouth. Conclusively, WFs work based on both pressure and pulsation, highlighting their technological sophistication and importance in modern dental care practices [15].

Comparison to traditional methods of interdental care

Studies show that 30% of the total adult population uses interdental aids, with floss being the preferred method of care [16,17]. Few data are available with regard to the frequency of use of all of the different aids and preferred methods of cleaning the interdental area. A Swedish study [18], assessing the use of different interproximal cleaning aids (dental floss, interdental brushes (IDBs), and toothpicks) daily, found that the preferred interdental care routine varied with respect to age group. Dental floss dominated in the younger age groups and IDBs in the two oldest groups. A US study [19] found that from the options of floss, wooden toothpicks, rubber tips, and IDBs, floss is the most used method of interdental care. Studies have recommended WFs for use in patients lacking manual dexterity those with fixed prostheses or those undergoing orthodontic treatment [20-22].

There were no population-based studies found that provide insight into the number of people currently using WFs, however, it is logical that this would vary, due to cultural, economic, and marketplace differences in the region. A Saudi Arabian study [23] showed that 114/240 (47.5%) of participants reported using WFs, which may not be representative of the entire population, as questionnaires were administered to patients undergoing fixed orthodontic treatment at a dental hospital, which could have led to selection bias. The same study found that 97/114 (85%) of participants stated that the WF assisted in interdental care and improved their gingival health. 

WFs versus traditional dental floss

Dental floss is a thin, flexible strand designed to slide between teeth and aid in the removal of plaque and food particles. Studies have emphasized its effectiveness in interdental plaque removal [24]. There are many types of floss, waxed, unwaxed, and specialized floss, which include variations such as silk floss and SuperFloss. These floss types appear to demonstrate the same efficacy [25,26]. A study by Akram [27] found that a WF was found to be more effective than dental floss for interdental care. The study found that the mean percentage reduction of the gingival index, plaque index, and bleeding on probing scores was higher in the group using a WF and toothbrushing compared to the group using only toothbrushing and the group using dental floss and toothbrushing. Abdellatif et al. [28] conducted a randomized controlled clinical trial comparing the plaque removal efficacy of WFs to traditional floss, highlighting the WF's effectiveness. A study by Mancinelli-Lyle et al. [29] demonstrated that WFs surpass traditional flossing in removing plaque and reducing gingival inflammation. Ng and Lim's overview [24] of interdental aids emphasized the efficacy of WFs, showcasing their ability to improve oral hygiene. Research by Xu et al. [30] demonstrated that adjunctive water flossing significantly reduces gingival inflammation compared to toothbrushing alone. This highlights the potential of WFs in contributing to overall gingival health. A clinical trial by Barnes et al. [31] revealed that WF use combined with a manual toothbrush was more effective at reducing bleeding and gingivitis than manual brushing and flossing. Notably, the group using a combination of water flossers and manual brushing experienced an almost two-fold increase in the reduction of bleeding compared to the group using a manual brush and dental floss. The study also found that WFs paired with a power toothbrush were more effective at reducing bleeding and more effective at reducing gingivitis than manual brushing and flossing. WFs and manual brushing removed plaque just as well as manual brushing and flossing on lingual surfaces, while WFs plus power brushing was more effective than manual brushing and flossing on facial surfaces of teeth.

WFs versus IDBs

IDBs have a handle and a small, narrow brush head with fine bristles, allowing users to access and clean tight spaces between teeth [32]. There is a dearth of information comparing the efficacy of WFs with IDBs. A study by Goyal et al. found that WFs are 56% more effective than IDBs for reducing the BOP of the entire mouth, 44% more effective in reducing facial BOP, 53% more effective in reducing interproximal BOP, and 41% more effective in reducing BOP in the interproximal facial region [33]. The study concluded that WFs are more effective than IDBs in reducing gingival bleeding over two weeks [34].

WFs versus interdental tape

Dental tape is characterized by its wider and flatter structure compared to traditional dental floss, making it suitable for individuals with larger interdental spaces or those who find conventional floss challenging to use [35]. While no direct studies specifically compare dental tape and WFs, general research supports the efficacy of both methods in interdental plaque removal. Individual preferences, ease of use, and oral health conditions should guide the choice between dental tape and WFs.

WFs versus electric flossers

An oscillating electric floss is a dental device that uses a motor to produce back-and-forth movements to clean teeth. The flosser element is stretched taut on a floss holder, which is mounted on a drive shaft that is rotationally oscillated by the motor [36]. There are currently no articles comparing the efficacy of WFs versus electric flossers in the management of periodontal disease.

WFs versus wooden toothpicks

There were no articles found that directly compare WFs with wooden toothpicks; however, WFs have many advantages over the use of wooden toothpicks. Toothpicks remove fibrous protein after meals but have a predisposition for trauma. A study concluded that other means of interdental care should be introduced, such as interdental brushing or flosser [37]. In some regions, various traditional methods such as teeth-cleaning sticks, twigs, and charcoal are still commonly used for interdental cleaning, which appear to share similar traits as mechanically cleaning with a wooden toothpick [38]. Toothpick use ranks poorly as an interdental cleaning aid [39-41].

WF with water alone versus WF with medicaments

Studies exploring the integration of medicaments with WFs have shown promising results. Edlund et al. [42] found that power-driven interdental aids, including WFs, significantly contributed to plaque removal when combined with appropriate medicaments. This highlights the potential of enhancing the benefits of water flossing by incorporating specific agents to address diverse oral health needs.

Ren et al. [43] demonstrated that oral irrigators with antimicrobial solutions effectively controlled dental plaque and gingival inflammation. This suggests the potential use of antimicrobial agents, such as chlorhexidine or essential oils, to augment the antimicrobial action of WFs. As adjuncts to toothbrushing, oral irrigators with anti-inflammatory solutions have shown promise in reducing clinical signs of inflammation. This indicates that medicaments with anti-inflammatory properties, such as nonalcoholic chlorhexidine or herbal extracts with anti-inflammatory effects, could be beneficial when used with WFs. Research by Talasani et al. [44] compared ozonated water with chlorhexidine mouth rinse, demonstrating that ozonated water was less effective in reducing plaque in gingivitis patients. Tecco et al. [45] explored the effects of an ozonated water irrigator on the plaque index, revealing a significant decrease in the BOP index over time. Further research is warranted to fully understand the nuanced benefits and applications of ozonated water in the context of WFs for comprehensive oral care.

Interdental care regimens can also be paired with fluoride solutions, to contribute to remineralization and strengthen tooth enamel [46, 47]. Research supporting the efficacy of fluoride-containing irrigants is limited, but the use of fluoride varnish in conjunction with WFs may provide additional fluoride benefits.

Combination of water flossing and traditional flossing methods

Incorporating both WFs and traditional flossing into one's oral care routine can provide a comprehensive approach to plaque removal and gingival health, ultimately contributing to improved overall oral hygiene. A combined approach ensures a more thorough removal of interdental plaque, as WFs effectively reach areas that traditional floss may miss [48].

WFs in the treatment of peri-implant diseases

The management of peri-implant disease is a critical aspect of maintaining oral health for individuals with dental implants. Tufts University conducted a study [49] emphasizing the effectiveness of the Waterpik® Water Flosser (Water Pik, Inc., Fort Collins, CO), finding it more effective for cleaning around dental implants than flossing, further supporting the role of WFs in preventing peri-implant disease. These findings suggest that incorporating WFs into oral hygiene routines may play a significant role in mitigating the risks associated with peri-implant diseases.

Future scope

The future scope of WFs in managing periodontal disease is highly promising, propelled by continuous research and technological advancements in oral healthcare. As these technologies evolve, WFs are expected to undergo further refinements in design and functionality, allowing for more individualized care. This means that future WFs may be tailored to meet specific patient needs, such as optimized protocols for targeted plaque removal in individuals with orthodontic appliances or dental restorations.

Moreover, the integration of smart technologies into WFs is anticipated to enable personalized oral health monitoring and guidance. This technological evolution could revolutionize home-based periodontal condition management, making it more effective and user-friendly. The increasing market size of WFs indicates a growing awareness and acceptance of these devices among consumers, which is likely to foster continued innovation in this field. This trend suggests that WFs will not only become more efficient in addressing the diverse needs of patients but will also play a crucial role in the evolution of periodontal disease management practices [50].

Challenges and limitations

 A major issue, as highlighted in our literature review, lies in the limited comparative evidence, and lack of systematic reviews regarding the efficacy of WFs in the management of periodontal disease. There have been studies, which show that there is little to no effect on the composition of subgingival plaque and reducing periodontal pockets [51,52]

Furthermore, concerns about bacterial colonization and the risk of cross-contamination have been raised during regular daily use of power-driven WFs [53]. Dental irrigators can cause transient bacteremia, with incidence correlated to oral hygiene and periodontal disease [54]. This may be an important consideration for people who are at risk of endocarditis. Understanding these challenges is crucial for both users and oral health professionals to ensure that WFs are used effectively and supplemented appropriately with other oral hygiene practices.

## Conclusions

In conclusion, current literature consistently supports the efficacy of WFs in the management of periodontitis and is likely the most effective means of interdental care. The findings of this review collectively suggest that WFs can serve as a valuable adjunct to conventional oral hygiene practices, contributing to improved periodontal health. The gentle yet effective nature of WFs makes them particularly suitable for individuals with periodontitis, offering an alternative that enhances plaque removal and gingival inflammation management. As the field continues to explore innovative approaches to periodontal care, integrating WFs into routine oral hygiene practices holds promise for promoting better periodontal health outcomes.
